# Skin Cells’ Protection Against UVA-Induced Changes in Co-Cultured Keratinocytes–Fibroblasts’ Proteome and Released Signaling Proteins by 3-*O*-Ethyl Ascorbic Acid

**DOI:** 10.3390/ijms27125551

**Published:** 2026-06-19

**Authors:** Agnieszka Gęgotek, Iwona Jarocka-Karpowicz, Magda Mucha, Elżbieta Skrzydlewska

**Affiliations:** Department of Analytical Chemistry, Medical University of Bialystok, Kilinskiego 1, 15-069 Bialystok, Poland; iwona.jarocka-karpowicz@umb.edu.pl (I.J.-K.); mmucha1106@gmail.com (M.M.); elzbieta.skrzydlewska@umb.edu.pl (E.S.)

**Keywords:** skin cells, 3-*O*-ethyl ascorbic acid, co-culture, proteomics, UVA radiation

## Abstract

UVA radiation affects communication between the cells that create the human skin. To prevent UVA-induced damage, there is a constant search for compounds protecting all skin cells and homeostasis in their communication. Therefore, the aim of this study was to evaluate the effect of 24 h incubation with 3-*O*-ethyl ascorbic acid (EAA; 150 µM) on the intracellular proteome of co-cultured keratinocytes and fibroblasts after UVA irradiation (total dose 15 J/cm^2^), and on the protein profiles released into the medium by both cell types. A proteomic approach (nanoHPLC/QOrbiTrap) allowed the identification of proteins significantly modified by UVA and EAA. In keratinocytes, UVA radiation enhanced expression of pro-inflammatory and pro-proliferative/keratinizing proteins and decreased expression of antiapoptotic and antioxidant proteins, while in fibroblasts, UVA radiation induced expression mainly of pro-inflammatory proteins, simultaneously decreasing levels of proteins involved in the antioxidant response and growth factors. Increased pro-inflammatory protein and decreased growth factor levels were also observed in the medium. EAA restored the levels of these proteins compared to control cultures. The results of this study show that EAA may protect epidermal and dermal cells by reducing levels of pro-inflammatory proteins, increasing antioxidant system activity in skin keratinocytes and fibroblasts, and normalizing intercellular signaling.

## 1. Introduction

UVA radiation (315–400 nm) accounts for up to 95% of the total UV radiation reaching the human body from sunlight. It can penetrate not only the epidermis but also the deeper layers of the skin [1]. Its action is described as multifaceted, especially in the context of skin cells, which primarily constitute its basic layers: keratinocytes in the epidermis and fibroblasts in the dermis. UVA is known to directly induce the generation of reactive oxygen species (ROS) and is a factor leading to photoinstability and phototoxicity in keratinocytes and fibroblasts [2]. By simultaneously reducing the cytoprotective activity of the transcription factor Nrf2 (nuclear factor erythroid 2-related factor 2), UVA impairs the antioxidant system in skin cells, leading to oxidative stress [3]. As a result, UVA also alters the metabolism of membrane phospholipids, leading to a significant shift toward oxidative reactions and an increase in lipid peroxidation products, including reactive aldehydes (e.g., 4-hydroxynonenal (4-HNE) and malondialdehyde (MDA)), as well as polyunsaturated fatty acids (PUFA) cyclization products, i.e., prostaglandins [4]. These molecules are known to primarily mediate pro-inflammatory signaling, which is why strong inflammatory reactions of the skin are observed after exposure to UVA radiation [5]. In consequence, UVA contributes to premature skin aging (photoaging) and even to the development of skin cancers [6]. Therefore, there is a constant search for compounds that could protect skin cells from the harmful effects of UVA radiation. In the context of its prooxidant action, these compounds should induce a strong antioxidant response. The most common, natural, easily available, well-described, and relatively safe to use is ascorbic acid (vitamin C). However, ascorbic acid has a number of limitations when applied to the skin. It requires an acidic environment for its biological effects, is easily degraded by sunlight, and, as a hydrophilic molecule, has difficulty penetrating the skin when applied topically, which is primarily composed of lipids and their metabolites [7]. Therefore, lipophilic derivatives of ascorbic acid, including 3-*O*-ethyl ascorbic acid (EAA), are increasingly used in skin preparations [8,9].

**Figure 1:**
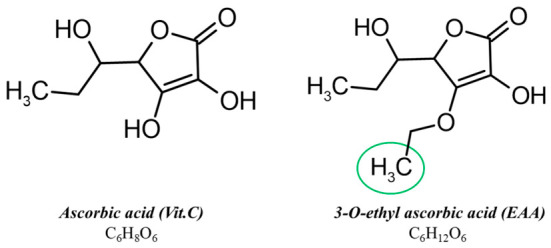
Comparison of the chemical structure of ascorbic acid and its derivative 3-*O*-ethyl ascorbic acid (EAA). The functional group differentiating these molecules is marked in green.

Similar to ascorbic acid, EAA is water-soluble; however, its structural modification by an ethyl group at the third carbon position makes it more stable [10] (Figure 1). The mentioned ethyl group protects the 3-OH group from ionization and, thus, the whole molecule from oxidation [11]. As a result, EAA has also different physicochemical properties than ascorbic acid, allowing it to penetrate the skin more effectively and making it more bioavailable [12]. Moreover, EAA has a stronger cytoprotective effect than ascorbic acid on UVA-irradiated skin cells [13]. EAA downregulates the expression/activity of tyrosinase and receptors (MC1R, TRP1/2) involved in melanin synthesis/translocation, thereby regulating skin color and significantly influencing the redox status of both pigmented and adjacent skin cells [14]. In vitro experiments on skin cells indicate that EAA acts not only as a ROS scavenger but may also activate kinase-based signaling (including p38 and PKC) and lead to Nrf2-dependent activation of the antioxidant system in keratinocytes [14,15]. Moreover, EAA significantly reverses the proteomic changes induced by UVA irradiation in keratinocytes [16]. This vitamin C derivative increases collagen biosynthesis in keratinocytes and dermal fibroblasts, as well as in reconstructed human epidermis exposed to UVB radiation [8]. Furthermore, EAA reduces the ROS levels, preventing oxidative modifications of other cellular components, including lipids, as evidenced by decreased lipid peroxidation (as reflected in lower MDA levels) in UVB-irradiated keratinocytes [17]. What is more, by decreasing the activity of phospholipid-metabolizing enzymes, including phospholipase A2 (PLA2), cyclooxygenases 1/2 (COX 1/2), and lipoxygenase 5 (LOX 5), EAA also regulates the enzymatic metabolism of phospholipids and PUFAs, resulting in the generation of endocannabinoids and eicosanoids, thus influencing prooxidant/anti-inflammatory signaling pathways involving these lipid mediators [17]. This suggests the possibility of a multifaceted protective effect of EAA on skin cells.

It is known that, after UVA exposure, cells in the deeper layers of the skin receive a lower radiation dose than those on the skin surface. However, cells in the deeper layers of the skin are also exposed to metabolites generated by more intensely irradiated cells, and the mechanism of action of EAAs on cells in different skin layers is not identical. Therefore, the aim of this study was to evaluate using MS-based approach the effects of 24 h incubation with EAAs (150 µM) on the intracellular proteome of keratinocytes and fibroblasts co-cultured after UVA irradiation (total dose 15 J/cm^2^), and on the protein profiles released into the medium by both cell types.

## 2. Results

**Figure 2:**
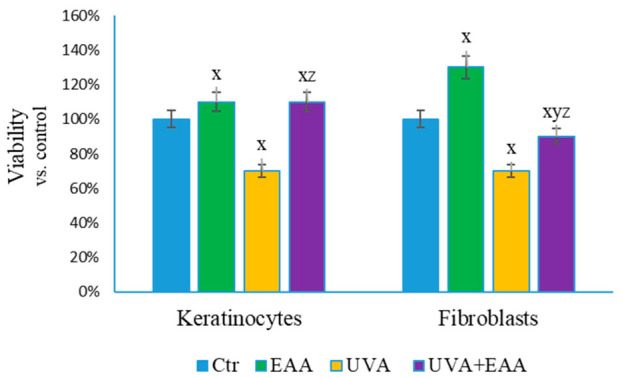
Viability measured by MTT assay of co-cultured keratinocytes and fibroblasts following exposure to UVA radiation [15 J/cm^2^] or/and treatment for 24 h with 3-*O*-ethyl ascorbic acid [150 µM]. Mean values ± SD are presented with statistically significant differences compared to the following: x—control cells; y—EAA treated cells; z—UVA irradiated cells. Abbreviations: Ctr: control; EAA: 3-*O*-ethyl ascorbic acid.

The findings of this study demonstrate that EAA at the used concentration increased cell viability by 10% and 30% compared to control cells for keratinocytes and fibroblasts, respectively. Moreover, it also significantly counteracted cell death induced by UVA radiation (Figure 2). Simultaneously, the results indicated also significant changes in the proteomes of UVA-irradiated and/or EAA-treated keratinocytes and fibroblasts in a co-culture model, as well as in the secreted protein profiles of these cells. Label-free semi-quantitative analyses identified and quantified the 1977 proteins, while 418, 494, and 168 were present in at least two samples from each group (keratinocytes, fibroblasts, and medium, respectively) and were therefore considered for further statistical analyses (Supplementary File S1).

**Figure 3:**
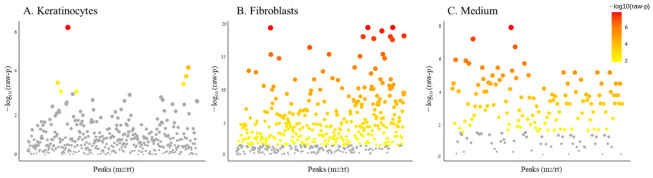
The one-way univariate analysis (ANOVA, *p* < 0.05) for proteins that were UVA-irradiated [15 J/cm^2^] or/and 3-*O*-ethyl ascorbic acid [EAA, 150 µM]-treated in co-culture keratinocytes (**A**) and fibroblasts (**B**), as well as in culture medium (**C**). The IDs and *p*-values for the statistically significant proteins are presented in Supplementary File S2.

**Figure 4:**
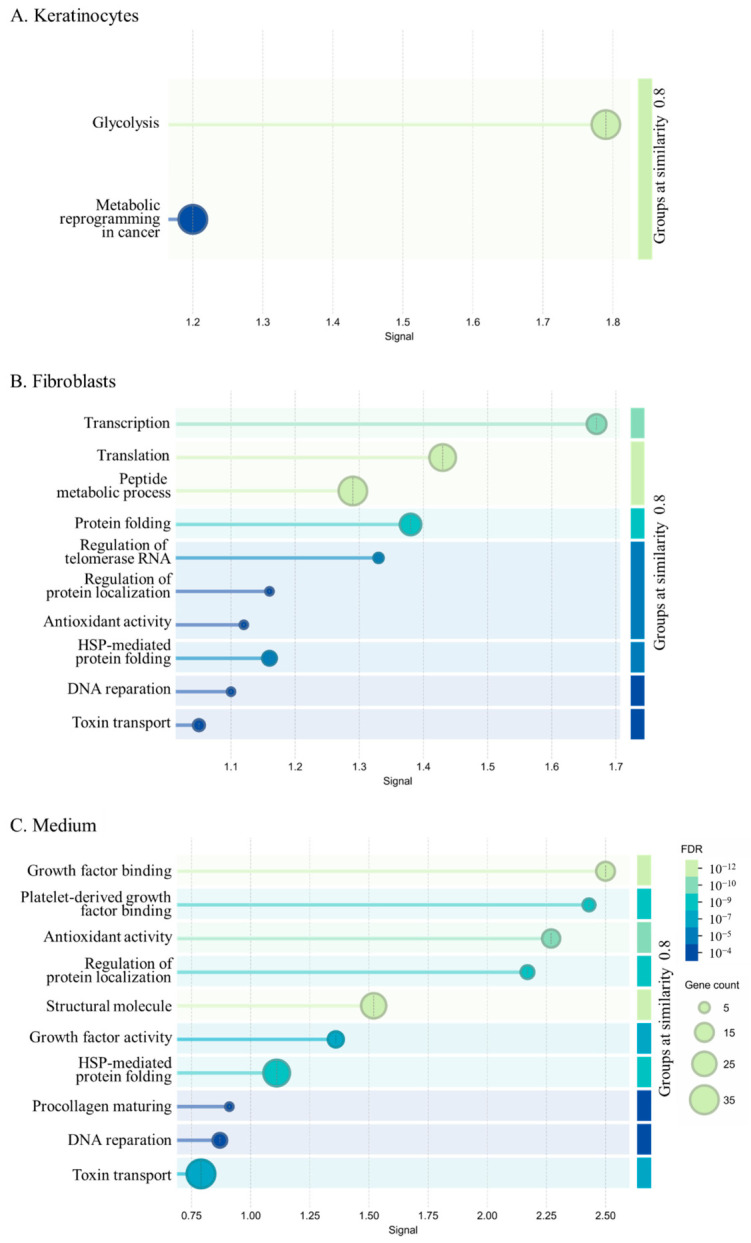
Biological functions of proteins significantly modified following UVA irradiation [15 J/cm^2^] or/and 3-*O*-ethyl ascorbic acid [EAA, 150 µM] treatment in co-cultured keratinocytes (**A**) and fibroblasts (**B**), as well as in culture medium (**C**).

ANOVA identified only seven significantly altered proteins in keratinocytes, with 331 in fibroblasts and 116 in the culture medium (Figure 3). Proteins indicated in keratinocytes as significantly modified were involved mainly in glycolytic processes and metabolic reprogramming (Figure 4A), while in the case of fibroblasts, the changes included a group of proteins associated with DNA/RNA repair or expression, protein/peptide folding, and antioxidant activity and toxin transport (Figure 4B). The proteins with altered levels in the medium were mainly signaling molecules, such as growth factors, but also molecules related to protein maturation/localization, DNA repair, antioxidant activity, toxin transport, and structural molecules (Figure 4C).

**Figure 5:**
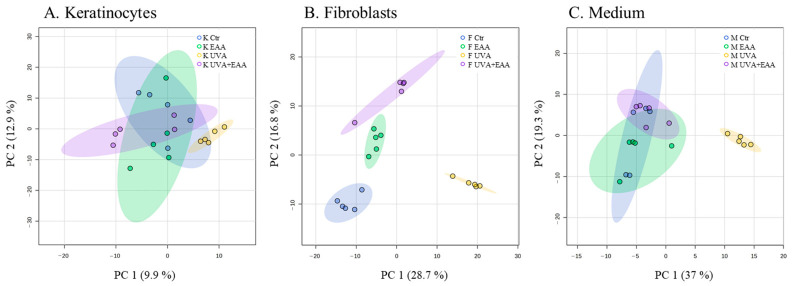
The results of principal component analysis (PCA) for proteins that were UVA-irradiated [15 J/cm^2^] or/and 3-*O*-ethyl ascorbic acid [150 µM]-treated in co-culture keratinocytes (**A**) and fibroblasts (**B**), as well as in culture medium (**C**). Abbreviations: Ctr: control; EAA: 3-*O*-ethyl ascorbic acid; PC: principal component.

**Figure 6:**
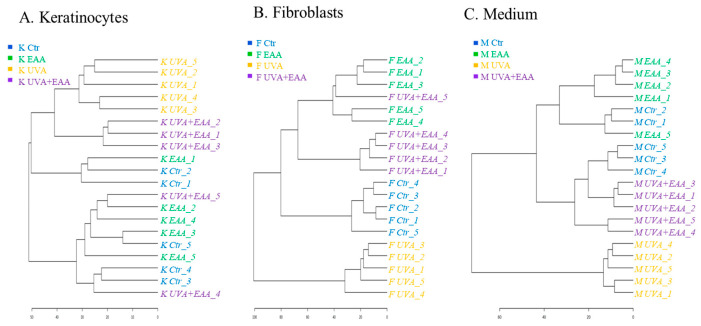
Dendrograms created for proteins that were UVA-irradiated [15 J/cm^2^] or/and 3-*O*-ethyl ascorbic acid [150 µM]-treated in co-culture keratinocytes (**A**) and fibroblasts (**B**), as well as in culture medium (**C**). Abbreviations: Ctr: control; EAA: 3-*O*-ethyl ascorbic acid.

Principal component analysis (PCA) indicated partial separation of the different experimental cell groups, especially in the case of fibroblasts, as well as in the culture medium (PC1: 28.7% and 37%, respectively) (Figure 5B,C). In both cases, the UVA-irradiated group was most separated in one dimension from the other samples, while the rest formed a rather close location to each other. In the case of keratinocytes, a greater dispersion of the samples was observed, but, as before, it was possible to partially separate the UVA-irradiated cells (PC1: 9.9%) (Figure 5A). Similar results were obtained for dendrograms, where UVA-irradiated groups always formed a separate branch, while the remaining samples formed branches to varying degrees (Figure 6).

**Figure 7:**
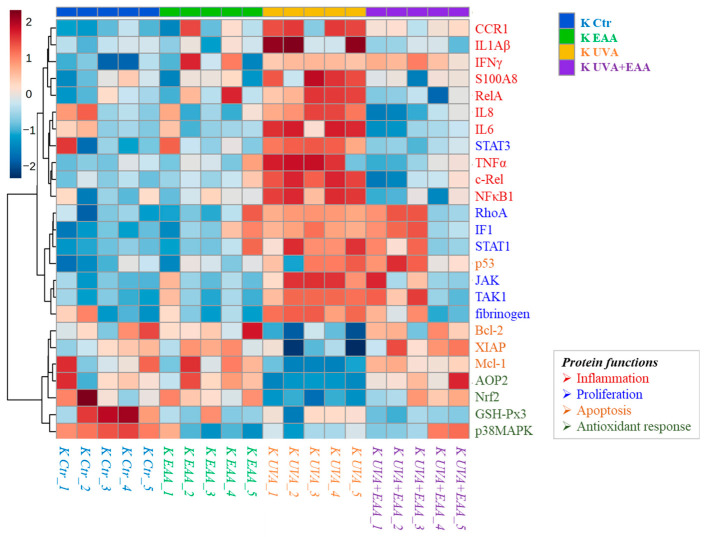
Heatmap created for top 25 modified proteins of keratinocytes following UVA radiation [15 J/cm^2^] or/and 3-*O*-ethyl ascorbic acid [150 µM] treatment in co-culture with fibroblasts. Validation of changes in the levels of three randomly selected proteins by Western blotting is provided in Supplementary File S3. Abbreviations: AOP: protein-derived antioxidant peptides; CCR1: C-C chemokine receptor type 1; Ctr: control; EAA: 3-*O*-ethyl ascorbic acid; GSH-Px: glutathione peroxidase; IF: intermediate filaments; IL: interleukin; JAK: Janus-activated kinase; MAPK: mitogen-activated protein kinase; Mcl: myeloid cell leukemia-1; NFκB: nuclear factor kappa-light-chain-enhancer of activated B cell; Nrf2: nuclear factor erythroid 2-related factor 2; STAT: signal transducers and activators of transcription; TAK: mitogen-activated protein kinase kinase kinase; TNF: tumor necrosis factor; XIAP: X-linked inhibitor of apoptosis protein.

**Figure 8:**
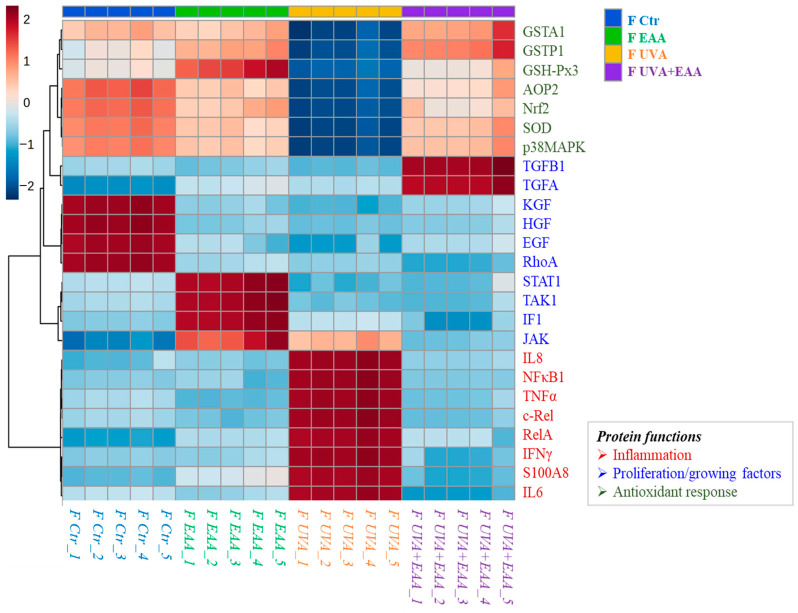
Heatmap created for top 25 modified proteins of fibroblasts following UVA radiation [15 J/cm^2^] or/and 3-*O*-ethyl ascorbic acid [150 µM] treatment in co-culture with keratinocytes. Validation of changes in the levels of three randomly selected proteins by Western blotting is provided in Supplementary File S3. Abbreviations: AOP: protein-derived antioxidant peptides; Ctr: control; EAA: 3-*O*-ethyl ascorbic acid; EGF: epidermal growth factor; GSH-Px: glutathione peroxidase; GST: glutathione S-transferase; HGF: hepatocyte growth factor; IF: intermediate filaments; IFN: interferon; IL: interleukin; JAK: Janus-activated kinase; KGF: keratinocyte growth factor; MAPK: mitogen-activated protein kinase; NFκB: nuclear factor kappa-light-chain-enhancer of activated B cell; Nrf2: nuclear factor erythroid 2-related factor 2; SOD: superoxide dismutase; STAT: signal transducers and activators of transcription; TAK: mitogen-activated protein kinase kinase kinase; TGF: transforming growth factor; TNF: tumor necrosis factor.

**Figure 9:**
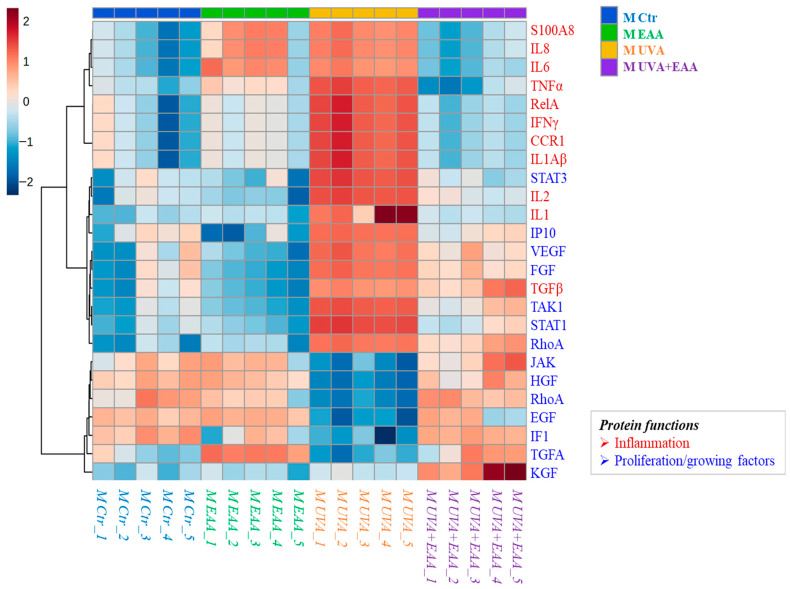
Heatmap created for top 25 modified proteins of medium from UVA-irradiated [15 J/cm^2^] or/and 3-*O*-ethyl ascorbic acid [150 µM]-treated in co-culture keratinocytes and fibroblasts. Validation of changes in the levels of three randomly selected proteins by Western blotting is provided in Supplementary File S3. Abbreviations: CCR1: C-C chemokine receptor type 1; Ctr: control; EAA: 3-*O*-ethyl ascorbic acid; EGF: epidermal growth factor; FGF: fibroblast growth factor; HGF: hepatocyte growth factor; IF: intermediate filaments; IFN: interferon; IL: interleukin; IP: interferon-gamma-inducible protein; JAK: Janus-activated kinase; KGF: keratinocyte growth factor; STAT: signal transducers and activators of transcription; TAK: mitogen-activated protein kinase kinase kinase; TGF: transforming growth factor; TNF: tumor necrosis factor; VEGF: vascular endothelial growth factor.

These observations were largely due to changes observed in the top 25 proteins with modified expression compared to the control cells/medium (Figure 7, Figure 8 and Figure 9). In the case of keratinocytes, despite substantial variation among the top 25 modified proteins, it was clear that UVA radiation significantly increased the expression of pro-inflammatory and pro-proliferative/keratinizing proteins and decreased the expression of antiapoptotic and antioxidant proteins. Treatment of keratinocytes with EAA significantly reduced UVA-induced pro-inflammatory proteins and restored the levels of antioxidant and antiapoptotic proteins to those of control cells (Figure 7). In fibroblasts, UVA radiation induced the expression of mainly pro-inflammatory proteins, simultaneously decreasing the levels of proteins involved in the antioxidant response and growth factors. Fibroblast treatment with EAA reversed the effects of UVA radiation, except for changes in growth factor expression (Figure 8). A similar effect was observed in the co-culture medium, with increases in pro-inflammatory proteins and decreases in growth factors. Medium supplementation with EAA restored the levels of these proteins compared to control cultures (Figure 9).

## 3. Discussion

Biomedical discoveries regarding the importance of healthy and properly functioning skin for the human body, as well as growing public awareness in this area, are increasingly directing research toward protecting the skin, including its cells, from harmful environmental factors, as well as metabolic regeneration after exposure to UV radiation, both as part of sunlight and used in cosmetic and medical applications. However, classic in vitro studies are most often based on the analysis of single cell lines and their response to UV radiation, as well as potentially protective or therapeutic compounds/preparations [18]. Human skin is a highly complex organ, and its proper functioning depends not only on the metabolism of individual cells within its layers but also on the interactions between them [19]. Therefore, co-culture models are increasingly used in studies of the metabolic responses of skin cells to external physicochemical conditions, enabling the acquisition of cellular responses more closely resembling real-world conditions [20,21]. It is known that both epidermal keratinocytes and dermal fibroblasts function differently in the presence of other cells, including influencing their metabolism. Keratinocytes are known to promote fibroblast proliferation and migration by releasing signaling molecules such as TGFβ [22,23], while fibroblasts also induce keratinocyte migration through cytokine biosynthesis, particularly in the presence of mechanical damage [24]. Furthermore, cells in a co-culture may respond differently to the same damaging factors. H_2_O_2_ has been found to reduce the viability of fibroblasts but not keratinocytes in co-culture [25], whereas in monocultures, H_2_O_2_ reduces the viability of both cell types [26,27]. Therefore, the aim of this study was to evaluate the effect of UVA radiation on co-cultured keratinocytes and fibroblasts and to analyze the potential protective/restorative effect of an ascorbic acid derivative, EAA, using a proteomic approach.

EAA is used as an ingredient in cosmetics, therefore it is also necessary to test its safety in everyday use. To date, EAA has been shown to cause serious side effects, such as allergic contact dermatitis [28]. However, this effect was observed in 7 of 160 subjects and was caused by a high dose of EAA (10%), which is higher than the dose commonly used in cosmetic preparations (~5%) [29]. The dose proposed in our experiment is subject to restrictive regulations and is approximately 0.003% (150 µM).

### 3.1. Changes in Pro-Inflammatory Protein Expression

The first symptom of excessive UVA exposure is inflammation [30]. This occurs due to both the overexpression of proteins involved in pro-inflammatory signaling and the increase in pro-inflammatory lipid mediators resulting from enhanced lipid oxidation in UVA-irradiated skin cells [31]. The study results clearly indicated that, in both cell lines, UVA radiation induces similar expression of pro-inflammatory proteins, including key cytokines IL-1α, IL-6, IFNγ, and TNFα. Moreover, not only are pro-inflammatory signaling molecules in skin cells upregulated but also proteins associated with NFκB transcription factor activity, including its subunits. A previous study showed that NFκB induction following UVA exposure of the skin leads to the production of pro-inflammatory cytokines, thereby further activating pro-inflammatory signaling [32]. Consequently, keratinocytes, as well as fibroblasts, release into the medium, signaling proteins that induce NFκB transcriptional activity in the neighboring cells, even those that build different layers of the skin [33]. However, the results observed in this study, i.e., the increase in various NFκB subunits, including p50, p52, RelA, and c-Rel, indicated divergent NFκB activity in canonical and non-canonical pathways [34]. On the one hand, heterodimers formed by RelA, c-Rel, and p50 or p52 induce the transcription of pro-inflammatory genes and the biosynthesis of pro-inflammatory proteins, while homodimers p50/p50 and p52/p52 inhibit the biosynthesis of pro-inflammatory proteins; however, in cooperation with the protein Bcl3, they activate the biosynthesis of proapoptotic proteins [35]. As a result, UVA-irradiated cells develop pro-inflammatory responses or enter the apoptotic pathway. This applies not only to keratinocytes, which receive the highest radiation dose, but also to deeper-lying fibroblasts, which, despite lower radiation exposure, show similar changes, likely due to cell–cell signaling.

In this study, EAA is a molecule characterized by anti-inflammatory properties, also in the context of skin cells [8]. It may act multidirectionally, simultaneously reducing oxidative stress and lipid peroxidation, resulting in lower concentrations of lipid pro-inflammatory mediators [17], as well as activating the phosphorylation of proteins involved in the NFκB pathway and silencing the expression of pro-inflammatory proteins [15,16]; however, there is not much literature data describing the possible mechanism of anti-inflammatory action of this molecule. The study results indicate that EAA decreases UVA-induced expression of pro-inflammatory factors by suppressing oxidative stress in skin cells and by reducing the expression of pro-inflammatory proteins, thereby significantly lowering the levels of released signaling molecules in the cellular medium. As a result, intercellular pro-inflammatory signaling is reduced, which, in vivo, may be crucial for protecting human skin against UVA radiation [36].

### 3.2. Changes in Antioxidant Protein Levels

UVA radiation also induces oxidative stress, directly contributing to the overproduction of ROS by activating endogenous photosensitizers and reducing the activity of the natural intracellular antioxidant system [3]. This is confirmed by the results obtained in this study for both co-cultured cell lines, keratinocytes and fibroblasts. In both cases, changes in the antioxidant protein profile focus on the reduced expression of antioxidant response enzymes, including SOD, CAT, and GSH-Px. Previous studies indicated that the activity of these enzymes is also reduced in skin cells after exposure to UVA radiation [31], which may result from both UVA-induced changes in their protein structure and decreased expression. Simultaneously, UVA decreases the level of Keap1, a cytosolic inhibitor of the Nrf2 transcription factor. This, combined with elevated MAPK levels, may lead to Nrf2 activation, prompting attempts to protect cells from chronic UV-induced oxidative stress [37].

EAA, as a derivative of ascorbic acid, is characterized by strong antioxidant properties, including the ability to capture ROS, as well as activate antioxidant enzymes and increase the level of non-protein antioxidants [16,38]. This study’s results indicate that EAA also prevents UVA-induced reductions in the expression of antioxidant enzymes (SOD, CAT, and GSH-Px) in both cell lines. On the other hand, EAA also prevents the UVA-induced decrease in Keap1 levels, thereby preventing the activation of the transcription factor Nrf2 and the induction of the synthesis of new antioxidant enzymatic/nonenzymatic proteins. This may be because the antioxidant effect of EAA is sufficient to protect cells from oxidative stress, and triggering the synthesis of new proteins would unnecessarily burden the cell energetically. Additionally, excessive and unregulated activation of Nrf2 is characteristic of cancer cells [39]. Moreover, in the list of significantly modified proteins in keratinocytes, proteins involved in glycolysis are also identified, suggesting that EAA can restore natural aerobic metabolism and protect skin cells from oxidative stress [40]. Following UVA irradiation, keratinocyte proliferation accelerates, making their metabolism more similar to that of cancer cells [41]. Therefore, EAA restoration of UVA-induced changes is important to protect skin cells against cancerous transformation, as is the case with other skin cells, such as melanocytes, which can transform into melanoma cells [42].

Interestingly, the culture medium and the cells also exhibit significantly altered protein expression, including antioxidant proteins. However, these proteins were not among the 25 most altered proteins. This may result from the random release of proteins into the medium by cells, as well as an attempt to maintain oxidative balance in the microenvironment of both fibroblasts and keratinocytes, as indicated in other skin cells, melanocytes, to prevent cancer transformation [43].

### 3.3. Changes in the Expression of Growth Factors

Our results indicate that the group of proteins whose changes following UVA exposure most differentiate the responses of keratinocytes and fibroblasts are growth factors and pro-proliferative cytokines. UVA-induced changes in keratinocytes lead to their overproliferation, while fibroblasts, following UVA irradiation, reduce the levels of EGF and TGF, attempting to stop keratinocyte proliferation, which may promote hyperkeratosis and, consequently, via the cMYB/PI3K-AKT pathway, contribute to delayed wound healing [44]. The literature data show that, in response to co-culture with keratinocytes, fibroblasts produce more HGF and KGF [45], as well as EGF, TGFβ, and IL-1α [46], to induce keratinocyte proliferation; however, this effect in our study is inhibited by UVA radiation. Increased keratinocyte proliferation following UVA exposure results from the epidermis’s predisposition to more intense exfoliation under harmful environmental conditions [47]. Moreover, prolonged oxidative stress can cause far-reaching changes in keratinocyte proliferation, even leading to the development of psoriasis [48]. All growth factors reported in this study to be induced by UVA radiation in keratinocytes are also upregulated in psoriatic skin lesions [49,50]. However, following UVA irradiation of co-cultured skin cells, the same proteins involved in the stimulation of keratinocyte proliferation are simultaneously decreased in fibroblasts, suggesting a role for fibroblasts in regulating keratinocyte proliferation [51]. The effect of EAA on keratinocytes is very clear—it strongly reduces the levels of growth factors, especially in cells after UVA irradiation, which may be due to reduced oxidative stress and the suppression of Nrf2 activity [14]. More complex changes are observed in fibroblasts: EAA increases the levels of selected growth factors in the absence of UVA irradiation, whereas after exposure to UVA irradiation, it reduces the levels of all identified growth factors. This dual action of EAA may lead to a self-protective fibroblast response, limiting fibroblast proliferation in order to avoid over-collagenization [52], simultaneously maintaining the appropriate number of biologically active fibroblasts in the dermis, ensuring constant production of the intercellular matrix [53], especially under UVA-induced prooxidant conditions, which accelerates the degradation of the intercellular matrix components and promotes photoaging [54]. Furthermore, EAA, both in UVA-irradiated and non-irradiated cells, increases the levels of proteins released by keratinocytes or fibroblasts that stimulate cell proliferation, growth, and maturation, thus counteracting UVA-induced changes.

On the other hand, EAA also restores the UVA-enhanced MMP levels in the medium from studied cells. MMPs, including MMP1 and MMP12, are upregulated in UV-irradiated skin fibroblasts, leading to accelerated aging [55,56]. Simultaneously, in UVA-irradiated keratinocytes, MMP2 and MMP9 are downregulated, showing an inverse response to UVA irradiation, depending on the cell line [57]. By preventing changes in MMP levels in the medium, EAA mutes UVA-induced degradation of components of the intercellular matrix, which is especially important in the dermis.

## 4. Materials and Methods

### 4.1. Cell Culture and Treatment

**Figure 10:**
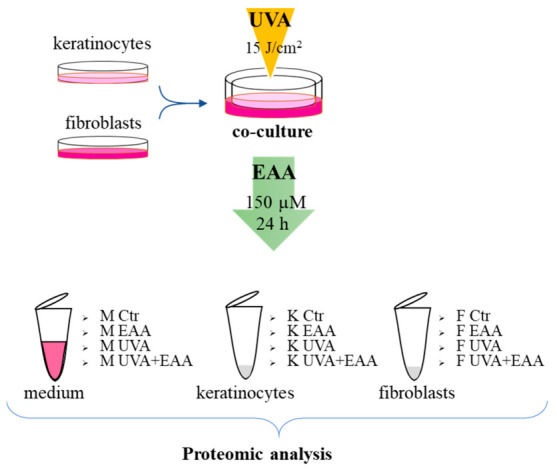
Scheme of the experiment conducted on co-cultured keratinocytes and fibroblasts: non-treated (control, Ctr) and UVA (15 J/cm^2^)-irradiated and/or treated for 24 h with 3-*O*-ethyl ascorbic acid (EAA, 150 µM).

Epidermal keratinocytes (CDD 1102 KERTr) and dermal fibroblasts (CCD 1112Sk) were obtained from the American Type Culture Collection (Manassas, VA, USA) with valid certificates of analysis and cultured according to the manufacturer’s protocols in a humid atmosphere of 5% CO_2_ at 37 °C. The complete growth medium for keratinocytes was Keratinocyte Serum-Free Medium supplemented with bovine pituitary extract (20 µg/mL) and human recombinant epidermal growth factor (0.1 ng/mL). The medium for fibroblasts was Dulbecco’s Modified Eagle Medium supplemented with 10% fetal bovine serum. To avoid bacterial contamination, penicillin/streptomycin (50 U/mL and 50 μg/mL, respectively) were added to both media. To create a co-culture, a 6-well plate with cell culture inserts with 0.4 μm pore size was used (Greiner Bio-One; Kremsmünster, Austria). Fibroblasts were seeded directly into the wells, and keratinocytes were seeded into the inserts. Cells in co-culture were exposed to UVA radiation (365 nm; Bio-Link Crosslinker BLX 365; Vilber Lourmat, Germany) in PBS (phosphate-buffered saline, 10 °C to avoid heat shock) at a dose of 15 J/cm^2^. The dose of UVA was chosen to ensure 70% of cells’ viability and to further ensure significant changes in lipid membranes and protein-based signaling associated with both pro-inflammatory and pro-apoptotic cell responses. Additionally, the used UVA radiation dose was similar to the estimated average daily exposure of skin cells to UVA radiation. Control cells, not exposed to UVA radiation, were cultured in parallel. To assess the effect of EAA, co-cultured cells were incubated for 24 h in medium containing 150 µM EAA, without supplementation with serum or growth factors. The concentration of EAA used to treat the co-cultured cells was selected as the highest dose that increased the viability of non-irradiated cells of both lines [13]. For cells’ treatment, the stock solution of EAA (10 mM) was prepared in PBS, and 15 µL was added per 1 mL of medium. The pH of the supplemented growing medium was 7.9, while the pH ensuring the highest EAA stability is 5.5 [58]. The pKa of EAA at room temperature is 7.7 [12]; therefore, the slightly deprotonated form of EAA may partially counteract the lowered pH observed during cell culturing. The experiment flow is shown in Figure 10.

Following incubation, the medium was collected for proteomic analyses. Keratinocytes in inserts and fibroblasts in wells were separated from each other, independently washed with PBS, and scraped on ice. Cell lysis was performed by sonication, and proteomic analysis was conducted on the supernatant obtained after centrifugation (15 min, 12,000× *g*, at 4 °C). Amicon columns (0.22 µm; Merck Millipore, Darmstadt, Germany) were used to increase the protein concentration in the culture medium. The total protein content in all samples was measured using a Bradford assay [59].

### 4.2. Viability Assay

For cell viability measurement, cells were seeded and treated in 6-well plates, with cell culture inserts with a 0.4 μm pore size (Greiner Bio-One; Kremsmünster, Austria), as indicated above. Following treatment, the medium from each well was removed, keratinocytes and fibroblasts were separated, and all cells were incubated with 0.25 mg/mL MTT solution in PBS for 40 min at a temperature of 37 °C. MTT was removed, and cells were lysed using dimethyl sulfoxide. The absorbance of formed formazan was read at 570 nm [13] using a Multiskan GO Microplate Spectrophotometer (Thermo Scientific, Waltham, MA, USA). The results were presented as a percentage of the value obtained for control cells.

### 4.3. Proteomic Analysis

Sample volumes containing 50 µg of proteins were taken for in-solution digestion. Then, proteins were denatured by urea (8 M), reduced with 1,4-dithiothreitol (10 mM), and alkylated using iodoacetamide (50 mM). Following fourfold dilution, samples were digested overnight (37 °C) with trypsin (Promega, Madison, WI, USA) in a ratio of 1:50 (trypsin:proteins). To stop the reaction, 0.1% formic acid was added to the samples [60]. The obtained peptide mixture was dried under inert gas and dissolved in 5% acetonitrile with 0.1% formic acid.

Peptides were separated using the high-performance liquid chromatography system Ultimate 3000 with a 150 mm  ×  75 µm PepMap RSLC capillary analytical C18 column (Dionex LC Packings, Dionex, Idstein, Germany) at a flow rate of 0.300 µL/min. Eluted peptides were analyzed using a Q Exactive HF mass spectrometer with an electrospray ionization source (Thermo Fisher Scientific, Bremen, Germany) operated in positive mode and data-dependent mode. Scans were conducted in the 200–2000 m/z range with a resolution of 120,000. In a subsequent scan, the top ten most intense ions were isolated, fragmented, and analyzed at 30,000 resolution. Tandem mass spectra were collected by Xcalibur software version 4.1 (Thermo Fisher Scientific, Bremen, Germany). The conditions for peptide analysis have been described in detail previously [61], and details of the analysis were described in the Supplementary File S1. Sample differences in the intensity of randomly selected peaks were validated using an Agilent 1290 Infinity II liquid chromatography system coupled to an Agilent 6475 liquid chromatography/quantum mass system (Agilent Technologies, Heilbronn, Germany), while changes in the levels of randomly selected proteins were validated using Western blot analysis. The method parameters and results of Western blotting are included in the Supplementary File S3.

### 4.4. Protein Identification and Label-Free Quantification

The data were analyzed using Proteome Discoverer 2.0 (Thermo Fisher Scientific, Seattle, WA, USA) against the UniProtKB-SwissProt database (taxonomy: Homo sapiens, release 2025-02). Default settings were applied for mass tolerance (10 ppm), MS/MS mass tolerance (0.02 Da), missed cleavages (up to 2), and dynamic modifications (cysteine carbamidomethylation and methionine oxidation). Protein label-free semi-quantification was performed according to the signal intensities of the precursor ions. Only proteins with at least three peptides longer than six amino acid residues and at least two unique peptides were taken for statistical analysis. Details of the analysis are described in the Supplementary File S4.

### 4.5. Statistical Analysis

Each experimental group variant was repeated 5 times in independent biological replicates. The results from individual protein label-free quantification were subjected to data imputation (missing values were replaced by 1/5 of the minimal positive values of their corresponding variables), assuming that the missing data are not random but represent low concentrations below the detection limit. To obtain the normal distribution, results from individual protein label-free semi-quantification were log-transformed, auto-scaled (mean-centered and divided by the standard deviation of each variable), and normalized by the sum of the protein intensities obtained for each sample using open-source software MetaboAnalyst 6.0 [62]. The same software was used for univariate analysis (one-way ANOVA, Fisher’s least significant difference (LSD) tests, false discovery rate < 5%), as well as for principal component analysis (PCA), dendrogram, and heatmap creation. Hierarchical clustering heatmap was created on normalized data with Euclidean distance measurement using ward method for clustering. For the designation of the top 25 differentially abundant proteins, ANOVA was applied as before. The sample distribution in PCA was computed using the Euclidean distance. Protein functions were determined using the Gene Ontology (GO) database [63], and functional enrichment visualization was performed in STRING 12.0 [64].

## 5. Conclusions

Since UVA radiation is one of the most intense and daily environmental harmful actors reaching human skin, there is constant searching for compounds that can restore/prevent changes in the metabolism of cells that build various skin layers, as well as maintain homeostasis in intercellular signaling, in order to ensure the continuity and good condition of the skin. The results of this study show that EAA may act as a regenerative compound, as it reduces levels of pro-inflammatory proteins, increases the activity of the antioxidant systems of skin cells (keratinocytes and fibroblasts), and normalizes intercellular signaling. Combining it with the strong penetrating properties of EAA and its higher stability than ascorbic acid, this compound may appear to be irreplaceable in the formulas of pharmaceuticals for reducing skin inflammation or skin care products. Moreover, the preliminary nature of the obtained results may indicate directions for further analysis in cosmetology or even skin pharmacology, because if studies on a large group of people provided positive results without side effects (depending on the concentrations used), it is possible that the protective/regenerative/anti-inflammatory effect of EAA could be used as a supporting effect for skin therapies. However, the use of EAA in only one concentration, which is the main limitation of the presented study, may give a quite narrow insight into the actual effects of this compound, which requires further analysis. Moreover, the nature of proteomic analysis with large amounts of data, limits mechanistic insight into observed changes. In the context of the data received, it requires complementary biochemical assays related to oxidative stress and antioxidant capacity, associated with the activity with key signaling proteins, such as Nrf2, NFκB, p53, JAK and STAT, including their phosphorylation level and nuclear translocation, to confirm the activation or inhibition of these pathways.

## Data Availability

The authors confirm that the data supporting the findings of this study are available within Supplementary Materials. The mass spectrometry proteomics data have been deposited to the ProteomeXchange Consortium via the PRIDE [65] partner repository with the dataset identifier PXD071840.

## Supplementary Materials

The following supporting information can be downloaded at https://www.mdpi.com/article/10.3390/ijms27125551/s1.

## Abbreviations

The following abbreviations are used in this manuscript:

4-HNE	4-hydroxynonenal
AKT	protein kinase B
ANOVA	analysis of variance
CAT	catalase
cMYB/PI3K	transcription factor MYB/phosphoinositide-3-protein kinase
COX	cyclooxygenase
c-Rel	cellular homolog of v-Rel
EAA	3-*O*-ethyl ascorbic acid
EGF	epidermal growth factor
GO	Gene Ontology
GSH-Px	glutathione peroxidase
HGF	hepatocyte growth factor
IFN	interferon
IL	interleukin
KGF	keratinocyte growth factor
LC/MS	liquid chromatography/mass spectrometry
LOX	lipoxygenase
LSD	least significant difference
MAPK	mitogen-activated protein kinase
MC1R	melanocortin 1 receptor
MDA	malondialdehyde
MMP	metalloproteinase
NFκB	nuclear factor kappa-light-chain-enhancer of activated B cells
Nrf2	nuclear factor erythroid 2-related factor 2
p38	protein 38
p50	protein 50
p52	protein 52
PBS	phosphate-buffered saline
PCA	principal component analysis
PKC	protein kinase C
PLA2	phospholipase A2
PUFA	polyunsaturated fatty acid
RelA	REL-associated protein A
ROS	reactive oxygen species
SOD	superoxide dismutase
TGF	transforming growth factor
TNF	tumor necrosis factor
TRP	transient receptor potential
