# Supplementary material for: Skin Cells’ Protection Against UVA-Induced Changes in Co-Cultured Keratinocytes–Fibroblasts’ Proteome and Released Signaling Proteins by 3-O-Ethyl Ascorbic Acid
Source: Int J Mol Sci. 2026 Jun 19;27(12):5551. doi: 10.3390/ijms27125551 (PMC13299177; doi:10.3390/ijms27125551)
Supplement: Supplementary file 1 [file ijms-27-05551-s001.zip › S3.pdf]

Supplementary File S3. Detailed description of proteomic results validation using Western blotting for cell lysates and medium of UVA irradiated [15 J/cm^2] or/and 3-O-ethyl ascorbic acid [150 μM] treated in co-culture keratinocytes and fibroblasts.

Methods

Western blotting

Following cells lysis, samples (cell lysates or medium) containing 40 μg of proteins were mixed with sample loading buffer (Laemmle buffer containing 5% 2-mercaptoethanol), heated at 95°C for 7 min, and separated on 10% Tris-Glycine SDS-PAGE gels. Following electrophoresis, proteins from gels were transferred to nitrocellulose membrane and were incubated for 1 hour with 5% blocking buffer (skim milk in TBS-T buffer with 5% Tween 20) [doi:10.3791/2359]. Primary antibodies against NFκB (p52), TNFα, Bcl2, p53 (Sigma-Aldrich, St. Louis, MO, USA)(host: mouse), Nrf2, IL2, IL6 (Invitrogen, Waltham, MA, USA)(host: rabbit) were used at a concentration of 1:1000 overnight. β-actin (host: mouse)(Sigma Chemical Co., St. Louis, MO, USA; cat. no. A2228) estimation was used as an internal control. Secondary mouse/rabbit antibodies were conjugated with alkaline phosphatase (Sigma-Aldrich, St. Louis, MO, USA; cat. no. A3687) and were used at a concentration of 1:2000 for 2 hours. Bands were visualized using the BCIP/NBT system (Sigma-Aldrich, St. Louis, MO, USA; cat. no. S006).

Results

<table>
<tr><td></td><td></td><td>Ctr_1</td><td>EAA_1</td><td>UVA_1</td><td>UVA+EAA_1</td><td>Ctr_2</td><td>EAA_2</td><td>UVA_2</td><td>UVA+EAA_2</td><td>Ctr_3</td><td>EAA_3</td><td>UVA_3</td><td>UVA+EAA_3</td></tr>
<tr><td rowspan="2">Keratinocytes</td><td>Bcl2</td><td colspan="7">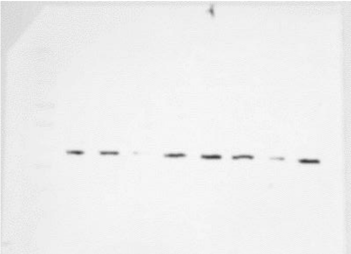</td><td colspan="5"></td></tr>
<tr><td>NFκB</td><td colspan="7">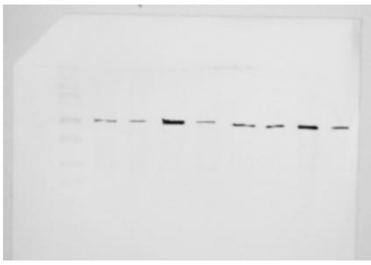</td><td colspan="5"></td></tr>
</table>

	p53	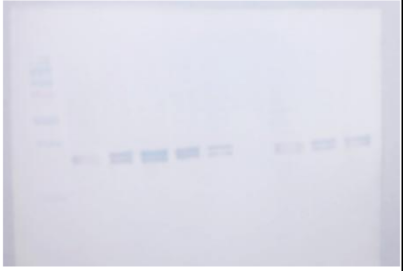	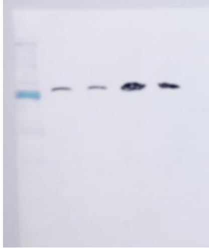
	β-actin	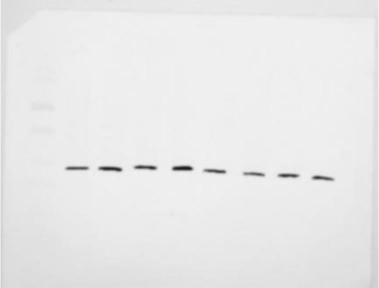	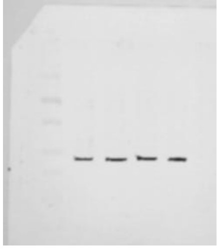
Fibroblasts	TNFα	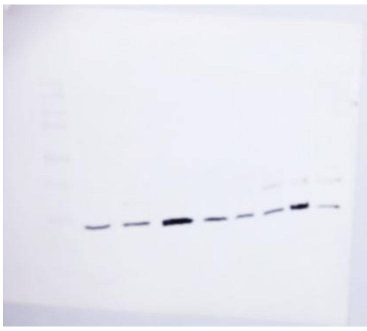	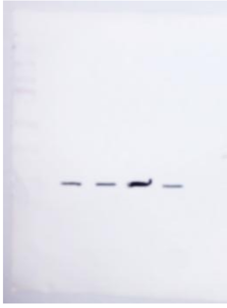
	NFκB		
	Nrf2	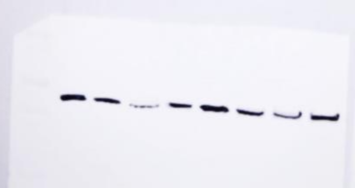	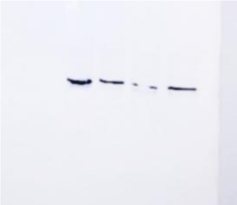

	β-actin	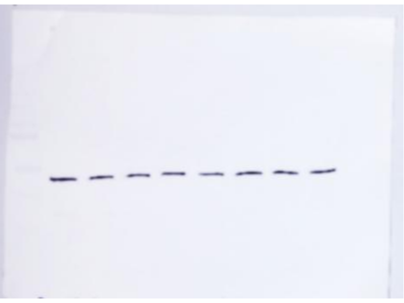	
Medium	TNFα		
	IL2	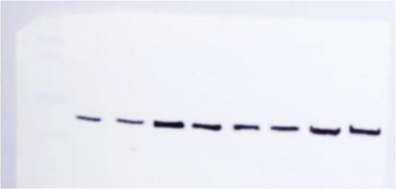	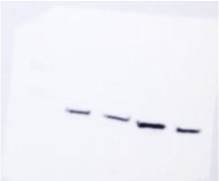
	IL6	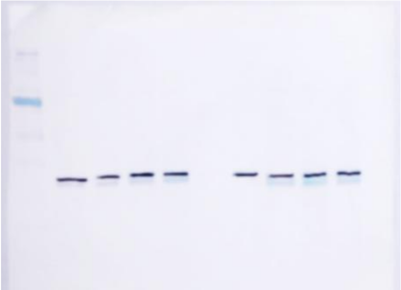	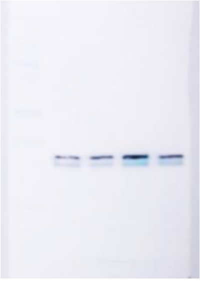
