# Supplementary material for: Skin Cells’ Protection Against UVA-Induced Changes in Co-Cultured Keratinocytes–Fibroblasts’ Proteome and Released Signaling Proteins by 3-O-Ethyl Ascorbic Acid
Source: Int J Mol Sci. 2026 Jun 19;27(12):5551. doi: 10.3390/ijms27125551 (PMC13299177; doi:10.3390/ijms27125551)
Supplement: Supplementary file 1 [file ijms-27-05551-s001.zip › S4.pdf]

Supplementary File S4. The details of the method parameters for proteins identification and quantification used for medium and cell lysates of UVA irradiated [15 J/cm^2] or/and 3-O-ethyl ascorbic acid [150 µM] treated in co-culture keratinocytes and fibroblasts.

A. ***Method of UHPLC Dionex UltiMate3000 RSLCnano System*** (Dionex, Idstein, Germany) with a 150 mm x 75 µm PepMap RSLC capillary analytical C18 column (Dionex, LC Packings)

Instrument Setup

UV.TimeConstant	0.12 [s]
UV.Data_Collection_Rate	10.0 [Hz]
Sampler.Temperature.Nominal	5.0 [°C]
PumpModule.LoadingPump.Pressure.LowerLimit	0 [bar]
PumpModule.LoadingPump.Pressure.UpperLimit	500 [bar]
PumpModule.NC_Pump.Pressure.LowerLimit	2 [bar]
PumpModule.NC_Pump.Pressure.UpperLimit	800 [bar]
PumpModule.NC_Pump.MaximumFlowRampUp/Down	0.300 [µl/min^2]
ColumnOven.Temperature.Nominal	40.0 [°C]
UV.UV_VIS_1.Wavelength	214 [nm]

Time 0.000

Inject Preparation

Time 3.000

PumpModule.NC_Pump.Flow.Nominal	0.300 [µl/min]
PumpModule.NC_Pump.%B.Value	4.0 [%]

Time 40.000

PumpModule.NC_Pump.Flow.Nominal	0.300 [µl/min]
PumpModule.NC_Pump.%B.Value	55.0 [%]

Time 44.000

PumpModule.NC_Pump.Flow.Nominal	0.300 [µl/min]
PumpModule.NC_Pump.%B.Value	90.0 [%]

Time 50.000

PumpModule.NC_Pump.Flow.Nominal	0.300 [µl/min]
PumpModule.NC_Pump.%B.Value	4.0 [%]

Time 60.000

PumpModule.LoadingPump.Flow.Nominal	5.000 [µl/min]
PumpModule.NC_Pump.Flow.Nominal	0.300 [µl/min]
PumpModule.NC_Pump.%B.Value	4.0 [%]

Stop Run

B. ***Method of Q Exactive HF*** with an electrospray ionization source (ESI) (Thermo Fisher Scientific, Bremen, Germany)

Overall method settings

Global Settings

Chrom. peak width (FWHM)	15 s

Time

Method duration	60.00 min

Full MS/dd-MS^2 (TopN)

General

Runtime	0 to 60 min
Polarity	positive

In-source CID	0.0 eV
Default charge state	2

Full MS

Microscans	1
Resolution	120,000
Maximum IT	100 ms
Number of scan ranges	1
Scan range	200 to 2000 m/z
Spectrum data type	Profile

dd-MS²/dd-SIM

Microscans	1
Resolution	30,000
Maximum IT	50 ms
TopN	5
Isolation window	4.0 m/z
Isolation offset	0.0 m/z
Scan range	200 to 2000 m/z
Spectrum data type	Profile

dd Settings

Intensity threshold	2.0e4
Multiple charge states	all
Peptide match	preferred
Exclude isotopes	on
Dynamic exclusion	10.0 s

C. ***Method of data processing using Proteome Discoverer 2.0*** (Thermo Fisher Scientific, Bremen, Germany)(searched against the UniProtKB - SwissProt database (taxonomy: Homo sapiens, release 2025-02))

Workflow of processing step

Processing node 1: Spectrum Selector

General Settings:	
- Precursor Selection:	Use MS1 Precursor
- Use New Precursor Reevaluation:	True
- Use Isotope Pattern in Precursor Reevaluation:	True
Spectrum Properties Filter:	
- Min. Precursor Mass:	350 Da
- Max. Precursor Mass:	5000 Da
- Total Intensity Threshold:	0
- Minimum Peak Count:	1
Scan Event Filters:	
- MS Order:	Is Not MS1
- Min. Collision Energy:	0
- Max. Collision Energy:	1000
- Scan Type:	Is Full
Peak Filters:	
- S/N Threshold (FT-only):	1.5
Replacements for Unrecognized Properties:	
- Unrecognized Charge Replacements:	Automatic
- Unrecognized Mass Analyzer Replacements:	ITMS
- Unrecognized MS Order Replacements:	MS2
- Unrecognized Activation Type Replacements:	CID
- Unrecognized Polarity Replacements:	+

- Unrecognized MS Res@200 Replacements:	60000
- Unrecognized MSn Res@200 Replacements:	30000
Precursor Pattern Extraction:	
- Precursor Clipping Range Before:	2.5 Da
- Precursor Clipping Range After:	5.5 Da

Processing node 2: MS Amanda

Input Data:	
- Protein Database:	Homo sapiens, release 2025-02
- Enzyme Name:	Trypsin
- Missed Cleavages:	2
- MS1 tolerance:	5 ppm
- MS2 tolerance:	0.02 Da
Dynamic Modifications:	
Dynamic Modification:	Carbamidomethyl
Substitution=	"H(3) C(2) N O" PositionType="Any" />
Dynamic Modification:	Carbamyl
Substitution=	"H C N O" PositionType="Any" />
Additional Settings:	
- Max No. of same modifs:	3
- Max No. of dynamic modifs:	4
- Ion Settings:	b,y
- Max. Rank:	5
- Max number of same neutral losses (H2O, NH3):	1
- No. of considered NLs (modifications):	2
- Perform deisotoping:	True
- Use monoisotopic mass:	True

Processing node 3: Percolator

Input Data:	
- Maximum Delta Cn:	0.05
Decoy Database Search:	
- Target FDR (Strict):	0.01
- Target FDR (Relaxed):	0.05
- Validation based on:	q-Value

Processing node 5: Event Detector

General Settings:	
- Mass Precision:	2 ppm
- S/N Threshold:	1

Processing node 6: Precursor Ions Area Detector

No parameters

<u>Workflow of consensus step</u>

Processing node 1: PSM Grouper

Peptide Group Modifications:	
- Site Probability Threshold:	75
Display Options:	
- Modification Sites Shown:	Best Position

Processing node 2: Peptide Validator

General Validation Settings:	
- Validation Mode:	Automatic (Control peptide level error rate if possible)
- Target FDR (Strict) for PSMs:	0.01
- Target FDR (Relaxed) for PSMs:	0.05
- Target FDR (Strict) for Peptides:	0.01
- Target FDR (Relaxed) for Peptides:	0.05

Specific Validator Settings:
- Validation Based on: q-Value
- Use Concatenated FDR Calculation for PSM Level FDR Calculation Based on Score: False
- Reset Confidences for Nodes without Decoy Search (Fixed score thresholds): False

Processing node 3: Peptide and Protein Filter
Peptide Filters:
- Peptide Confidence At Least: Medium
- Keep Lower Confident PSMs: False
- Minimum Peptide Length: 6
- Remove Peptides Without Protein Reference: False
Protein Filters:
- Minimum Number of Peptide Sequences: 1
- Count Only Rank 1 Peptides: False
- Count Peptides Only for Top Scored Protein: False

Processing node 4: Protein Scorer
No parameters

Processing node 5: Protein Grouping
Protein Grouping:
- Apply strict parsimony principle: True

Processing node 6: Protein FDR Validator
Confidence Thresholds:
- Target FDR (Strict): 0.01
- Target FDR (Relaxed): 0.05

Processing node 7: Peptide and Protein Quantifier
Ratio Calculation:
- Minimum Quan Value Threshold: 0.0001
- Replace Missing Quan Values With Minimum Intensity: False
- Reject All Quan Values If Not All Quan Channels Are Present: False
- Maximum Allowed Fold Change: 100
- Use Ratios Above Maximum Allowed Fold Change for Quantification: False
- Create Separate Quan Columns: False
Ratio Calculation for Precursor Quan:
- Use Single-Peak Quan Channels: False
Ratio Calculation for Reporter Quan:
- Apply Quan Value Corrections: True
- Co-Isolation Threshold: 100
Protein Quantification:
- Use Only Unique Peptides: True
- Consider Proteins Groups for Peptide Uniqueness: True
- Top N Peptides Used for Area Calculation: 3
Normalization:
- Experimental Bias Correction: None
- Minimum Ratio Count for Median Normalization: 20
- Manual Normalization Factor: 1
Quan Ratio Distributions:
- 1st Fold Change Threshold: 2
- 2nd Fold Change Threshold: 4
- 3rd Fold Change Threshold: 6
- 4th Fold Change Threshold: 8
- 5th Fold Change Threshold: 10
